# Pharmacal Failings

**Published:** 1880-12

**Authors:** 


### Editorial.

### PHARMACAL FAILINGS.

THE medical journals have of late had a good deal to say of our brethren of the "Pestle and Mortar," which has not been complimentary to them. Indeed, pharmacists as a class have been represented as guilty of the vices of "counter-prescribing,"

"substituting" and "repeating," and ready to avail themselves of every opportunity to take the bread out of the mouths of their superiors, the doctors. To correct these abuses, and to humble these "ignorant upstarts," it is proposed that each doctor should be his own druggist and carry and dispense his own medicines.

Now, we have reason to believe that the charges made are true. That it is the custom of druggists to repeat a prescription as often as it is called for, we do not think any of them will deny, and every physician knows, that serious injury to the physical and moral welfare of the patient often results from such unauthorized renewals, of medicines designed only for temporary employment under the observation of a prudent prescriber.

The practice of substitution of drugs in making up prescriptions, or the omission of an ingredient which the druggist may not have in stock, through negligence or want of capital, is a *crime* which deserves the severest censure. This practice places the life of the patient and the reputation of the physician at the mercy of a druggist or his clerk. We believe that this is more often done than physicians are aware of. Our cities abound with cheap drug stores, managed by men of little capital and less experience, and the temptation is great to substitute cheap for expensive drugs—Cinchona for Quinine for example—and thereby to increase their profit at the same time to supply the patient with the medicine at a less price than demanded by an honest competitor; or, if, when compounding prescriptions in which standard formulæ are called for, the druggist puts in some diluted medication of his own, under the same name, both patient and prescriber are again the victims of his dishonesty.

Another of the "ways that are dark" and yet inviting with the lure of gain, to some pharmacists, is the taking upon themselves the office of the physician and prescribing for persons over the counter. In this they are guilty of bad faith and business injustice to the physician, and dishonesty toward themselves and the patient, in claiming a knowledge which they do not possess.

Now, while there is no doubt about the truth of these charges as to individuals, it is as unjust to accuse pharmacists as a body of these practices, as it would be for them to accuse us of the infamies which we can not deny are practiced by individuals in our own ranks. From the experience of a good many years we are of the opinion that there are about the same proportion of honorable and upright men in the two professions.

The better class of pharmacists are as active and earnest in their efforts to rectify abuses, and to obtain adequate laws for the protection of themselves and the public, as are the doctors; but instead of recognizing this effort, many medical men look only at the injustice done them, and as a remedy propose to dispense with the dispensers.

This apparently simple remedy is more simple than wise or practical. It would in effect be, an attempt to stem the tide of progress. Such a plan was in vogue when medical knowledge was confined to very narrow limits. To-day the immense accumulation of knowledge as to disease and its treatment, and the enrichment of our materia medica by the labors of chemists and pharmacists, has necessitated a division of labor, and the progress of scientific medicine depends upon the faithful efforts of workers in each department: any attempt to combine the "art of prescribing and the art of dispensing," must result in a loss of knowledge and progress in both departments.

From a business point of view the argument that the evils would be corrected by dispensing our own medicines, is equally fallacious. It is based upon the idea that druggists are dependent upon physicians for their support—on the contrary, the majority of them make their chief profit from the sale of drugs and fancy goods, the dispensing of prescriptions being a secondary and comparatively unimportant part of their business, and it is just this class who are guilty of the practices condemned, and who are indifferent to our threats to put an end to their vocation, if they do not mend their ways.

In point of fact, the only ones whom the adoption of such a policy would injure are those whom we all honor. The men who are disposed to keep pharmacies and not general fancy goods stores, who conduct their business with scrupulous honesty and a just appreciation of their professional duties. Men of this class can not be accused of counter-prescribing or substituting; and a prescription is not repeated if a request to that effect accompanies the recipe.

After all, are these good fellows so entirely at our mercy? True, they are largely dependent upon our good will and patronage; but if we withdraw that, and turn druggists ourselves, can they not almost as easily turn doctors, and if the opprobrium which attachés itself, at least in the cities, to practicing physicians who are also druggists, is removed, will not the accomplished pharmacist who graduates in medicine have a decided advantage over the younger medical men in the race for professional success?

During the last few years pharmacy has assumed a position which fairly entitles it to rank as a profession, and the best interests of the medical profession are to be advanced, not by ignoring and decrying the value of pharmacy to medicine, but by encouraging the growth of a professional spirit among the Pharmacists, and by aiding them in their efforts to rectify abuses by legislative enactment. Meanwhile the physician must protect himself and his patients by exercising a strict oversight over the preparation of his prescriptions, allowing them to be dispensed only by pharmacists whom he knows to be competent and reliable; by *exposing* and *absolutely refusing* to allow his prescriptions to be filled by any druggist who has been detected in *substituting*, or who is known to be guilty of the minor offenses of counter-prescribing, etc.; by warning his patients of the dangers they incur in seeking to get their medicines at the *cheapest*

place, and by showing the same consideration, justice and fair play to the faithful apothecary that he has a right to expect from him.

